# Rhabdomyolysis as a manifestation of a metabolic disease: a case report

**DOI:** 10.5935/0103-507X.20170016

**Published:** 2017

**Authors:** Marta Sousa Moniz, Maria Inês Mascarenhas, Carlos Escobar, Pedro Nunes, Clara Abadesso, Helena Loureiro, Helena Almeida

**Affiliations:** 1 Unidade de Cuidados Intensivos Pediátricos, Departamento de Pediatria, Hospital Prof. Doutor Fernando Fonseca, E.P.E - Amadora, Portugal.

**Keywords:** Oxidation-reduction, Renal insufficiency, Rhabdomyolysis/diagnosis, Rhabdomyolysis/etiology, Metabolic diseases, Case reports

## Abstract

Rhabdomyolysis is a process of muscle destruction that can present with varying clinical manifestations. In pediatric patients, its main etiology is infectious diseases. We present a previously healthy adolescent who was admitted to our emergency department with a four-day history of myalgia, muscle weakness and dark urine. At presentation, she was dehydrated. Blood analysis revealed acute renal failure and increased muscular enzymes.

She was transferred to our pediatric intensive care unit. Medical therapies for correction of dehydration and the ionic and metabolic consequences of renal failure were performed. Due to oliguria, renal replacement therapy was initiated. An etiological investigation revealed a beta-oxidation defect. Metabolic diseases are a known cause of rhabdomyolysis. Muscular destruction should be diagnosed early in order to avoid its potential consequences. Generally, the treatment of rhabdomyolysis is conservative, although in some situations, a more invasive approach is needed.

## Abstract (pt)

A rabdomiólise é um processo de destruição muscular com manifestações clínicas variáveis. Em pacientes pediátricos, tem como principal etiologia as doenças infecciosas. Apresentamos o caso de uma adolescente previamente saudável, que foi admitida ao nosso pronto-socorro com histórico de 4 dias com mialgia, fraqueza muscular e urina escura. Na avaliação inicial, apresentava-se desidratada. Os exames de sangue revelaram insuficiência renal aguda e aumento de enzimas musculares. A paciente foi transferida para nossa unidade de terapia intensiva pediátrica. Foi realizado tratamento clínico para correção da desidratação e das consequências iônicas e metabólicas da insuficiência renal. Em razão da oligúria, deu-se início à terapia de substituição renal. A investigação etiológica revelou um defeito da betaoxidação. Sabe-se que doenças metabólicas podem provocar rabdomiólise. A destruição muscular deve ser identificada precocemente, para evitar suas potenciais consequências. Em geral, o tratamento da rabdomiólise é conservador, embora em algumas situações seja necessária uma abordagem mais invasiva.

## INTRODUCTION

Rhabdomyolysis is characterized by muscle necrosis that results in the release of muscles constituents, including myoglobin, into circulation. Typically, rhabdomyolysis presents with myalgia, muscle weakness and dark urine. However, this triad of symptoms may not always be present in children. Clinical manifestations may range from an asymptomatic illness to a life-threatening condition with highly elevated enzymes, acute renal failure (ARF) and electrolyte disturbances.^(1,2)^ Commonly, creatine kinase levels are markedly elevated, and myoglobinuria may be present.^(1,2)^ In pediatric patients, infectious diseases are the main cause of rhabdomyolysis. However, other etiologies can be found and include trauma, exercise, drugs, toxins, and metabolic and electrolyte disorders.^(1-3)^ Inherited disorders of carbohydrate metabolism, mitochondrial respiratory chain enzyme deficiencies, disorders of fatty acid oxidation and deficiency of carnitine palmitoyltransferase are examples of metabolic diseases associated with rhabdomyolysis.

We present a case of a previously healthy adolescent that presented with massive rhabdomyolysis and was diagnosed with a disorder of fatty acid oxidation.

## CASE REPORT

A 14-year-old girl was admitted to our emergency department with a four-day history of generalized myalgia, muscular weakness and dark urine. On the day of admission, she noted much-reduced diuresis and had difficulty walking. During the previous days, the patient had been participating in a dance festival in hot conditions. There was no past history of muscle cramps or hospital admissions due to rhabdomyolysis. She was the only child of non-consanguineous parents.

At presentation, she was dehydrated. Her blood pressure was 120/60 (90^th^ percentile). Her muscle strengths in the proximal and distal muscles of the upper and lower extremities were 4/5. Deep tendon reflexes were normal, and there was no neurologic deficit. The remainder of her physical examination was normal. Her weight was 57kg, and her height was 165cm.

The laboratory evaluation revealed ARF with a blood urea nitrogen of 263mg/dL (range 19.3 - 44.9mg/dL), a blood creatinine of 9.59mg/dL (range 0.60 - 1.30mg/dL) and a glomerular filtration rate calculated according to the original Schwartz formula of 11.6mL/min/1.73m^2^. The blood gases showed metabolic acidosis (pH 7.30; HCO_3_ 17.7mmol/L; base excess - 7.9; lactate 1.4mmol/L). Blood biochemistries were as follows: sodium 129mmol/L (range 136 - 145mmol/L), potassium 6.12 (range 3.4 - 5.1mmol/L), ionized calcium 1.03mmol/L (range 1.13 - 1.32mmol/L), phosphorus 9.3mg/dL (3.1 - 5.5mg/dL), magnesium 1.8mg/dL (range 1.6 - 2.3mg/dL), myoglobin 28173mg/dL (range 9 - 82mg/dL), creatine kinase > 400,000UI/L (range 28 - 142UI/L), aspartate aminotransferase 3266UI/L (range 0 - 26), alanine aminotransferase 1310UI/L (range 19 - 44UI/L).

Because of massive rhabdomyolysis, the patient was admitted to our pediatric intensive care unit and was given intravenous fluid combined with diuretic therapy to reverse ARF, glucose and insulin therapy to correct hyperkalemia and calcium gluconate to prevent cardiac arrhythmias secondary to ion changes. During the first hours of admission, she presented anuria that was unresponsive to medical therapy. After eight hours of receiving supportive therapy, she was started on continuous veno-venous hemodiafiltration (Gambro Prismaflex^®^ System, Lisbon; Portugal).

A hemodialysis catheter (12F; triple lumen) was inserted in the right femoral vein. Hemodiafltration was performed using the hemofilter ST 60, and heparin was the anticoagulant chosen. The following initial settings were used: blood-pump 150mL/min, dialysate (Prismasol 4^®^) 1000mL/hr, pre-filter replacement solution (Prismasol 4^®^) 500mL/hr, post-filter replacement solution (Prismasol 4^®^) 500mL/hr and fluid removal 50mL/hr. During the first two days of therapy, the main problem with hemodiafiltration was easy coagulability of the hemofilter due to high levels of myoglobin in circulation. To overcome this problem, higher pre-dilution flow rates were used (maximum 1500mL/h). After 48 hours, she had asymptomatic hypophosphatemia of 2.7mg/dL that was corrected after adding phosphorous to the replacement solutions. On day 5, intermittent hemodialysis was started, and three sessions on alternate days were performed. A calcium antagonist was prescribed on day six due to worsening hypertension.

Signs and symptoms were controlled with medical treatment and renal replacement therapy. Creatinine kinase and myoglobin returned to normal values in two weeks. Her diuresis started to recover after the second day of therapy. At discharge, her renal function was recovering, and she had a glomerular filtration rate of 88.9mL/min/1.73m^2^.

Regarding the investigation of the underlying insult, an acylcarnitine analysis by tandem mass spectrometry of the patient's dried blood spot revealed a deficiency of very long-chain acyl-CoA dehydrogenase (VLCAD). A genetic study revealed the following mutations in compound heterozygosity of the VLCAD gene: p.P65Tfs*7 (c. 187_192insA) and p.R336H (c.1097G > A).

Frequent meals with carbohydrate-rich intake before exercise and restriction of long-chain fatty acids intake along with medium-chain fatty acid supplementation were recommended to prevent further attacks.

## DISCUSSION

We present the case of an adolescent diagnosed with VLCAD deficiency after a massive episode of rhabdomyolysis.

VLCAD is an enzyme of fatty acid oxidation found in the inner membrane of the mitochondria, and it catalyzes the first step of long-chain fatty acid beta-oxidation.^(4)^ Its deficiency is inherited as an autosomal recessive trait with an incidence of 1:31,500 births.

Depending on the age at presentation (neonatal, childhood or adulthood), this disease has different clinical manifestations.^(4)^ The neonatal-onset form manifests as liver failure and cardiomyopathy, and the childhood-onset form is associated with hypoketotic hypoglycemia. Our patient presented with the adulthood form, which is predominantly characterized by muscle complaints due to recurrent episodes of rhabdomyolysis triggered by prolonged exercise, infections or fasting.^(4)^

Although currently VLCAD deficiency is mainly diagnosed with the newborn screening card that has allowed for earlier diagnosis, different methods can be used for its diagnosis, such as chromatographic analysis of plasma fatty acids, tandem mass spectrometry analysis of acylcarnitines and organic acids analysis in urine.^(5,6)^ In Portugal, this disease has been systematically screened for in the neonatal screening program since 2006, and our patient was born prior to this date.

For VLCAD deficiency, there is no definitive therapy. Treatment consists of diet modifications and should include a diet low in very long-chain fatty acids with supplements of medium-chain triglycerides. Fasting and intense exercise should be avoided.^(5,6)^

Particularly, in the adult form, when a crisis is not prevented or interrupted in time, treatment should be directed toward reversing rhabdomyolysis and its complications. In children, ARF is a recognized complication of muscle destruction, and according to a recent study, it can occur in 4 - 5.8% of patients with rhabdomyolysis.^(7)^ Although the basic mechanism of ARF in rhabdomyolysis conditions is not fully understood, one proposal is that the myoglobin released from the destroyed muscle is the responsible for the renal damage. Renal constriction and ischemia, myoglobin cast formation in distal tubules and direct myoglobin cytotoxic action in the epithelial cells of the proximal tubules are probably the main mechanisms underlying ARF.^(8)^ Other potential complications of rhabdomyolysis include hyperkalemia that may lead to cardiac arrhythmias, metabolic acidosis and hypocalcemia, compartmental syndrome and asymptomatic disseminated intravascular coagulation.^(8)^

The major therapeutic interventions in rhabdomyolysis are conservative. Initial management should include aggressive fluid resuscitation and hydration in order to maintain adequate urine output and prevent ARF, early correction of potentially lethal electrolyte disturbances and correction of metabolic acidosis. Intravenous isotonic saline should be started as soon as possible after the onset of muscle injury and continued until the injury has resolved. Although there is no clear clinical evidence for its effectiveness, a forced alkaline diuresis may be performed with bicarbonate administration in order to reduce the renal toxicity of heme.^(2,9)^ The precipitation of calcium phosphate and appearance of hypocalcemia should be monitored closely.^(9)^ The best regimen and rate of bicarbonate administration are unknown.^(9)^ Fluids should be carefully given once ARF and oliguria are present in order to avoid non-cardiogenic pulmonary edema. Although renal replacement therapy is rarely needed in rhabdomyolysis, it should be considered when there is severe and resistant hyperkalemia, persistent metabolic acidosis, uremia and ongoing ARF despite conservative treatments.^(8)^ Daily hemodialysis or continuous renal replacement therapy are both options that allow for gradual removal of solutes and potassium correction.^(10)^ However, both have limited ability to remove myoglobin from circulation due to the high molecular weight of myoglobin. Recently, based on case series descriptions, some authors have proposed performing CRRT with hyper-permeable membranes (e.g., super high-flux membranes) that allow for removal of molecules with higher molecular weights (cut-off 60,000 Da), such as myoglobin and cytokines.^(11)^ However, these membranes are associated with excessive albumin loss. Our patient used a standard filter, which had the main problem of easy coagulability in the first few days of treatment. This problem was attributed to the high circulating levels of myoglobin that obstructed the membrane pores. Peritoneal dialysis is inadequate to remove the large solute load in patients with rhabdomyolysis. Other complications of rhabdomyolysis, such as compartment syndrome, should be monitored, and surgical decompression should be performed as soon as needed. The treatment of disseminated intravascular coagulation is mainly supportive and depends on the overall treatment of rhabdomyolysis.

## CONCLUSION

Very long-chain acyl-CoA dehydrogenase deficiency is a disease that has the worst prognosis when it is diagnosed in the newborn period. The adult form has a good prognosis if rhabdomyolysis is avoided. Once in advanced stages, rhabdomyolysis can lead to life-threatening complications.
